# 9,9-Dimethyl-9,10-dihydroanthracene

**DOI:** 10.1107/S1600536811033526

**Published:** 2011-08-27

**Authors:** B. P. Siddaraju, Jerry P. Jasinski, James A. Golen, H. S. Yathirajan, C. R. Raju

**Affiliations:** a Department of Studies in Chemistry, University of Mysore, Manasagangotri, Mysore 570 006, India; b Department of Chemistry, Keene State College, 229 Main Street, Keene, NH 03435-2001, USA; c Department of Studies in Chemistry, University of Mysore, Bangalore 560 064, India; d Department of Chemistry, PES College of Science, Mandya 571 401, India

## Abstract

In the title compound, C_16_H_16_, the central benzene ring adopts a boat conformation, with a dihedral angle of 34.7 (9)° between the mean planes of the two fused benzene rings. The two methyl groups at the apex of the central benzene ring are in axial and equatorial conformations. The crystal packing is stabilized by weak C—H⋯π inter­molecular inter­actions.

## Related literature

For analytical applications of anthrone, see: Trevelyan (1952 ▶). For related structures, see: Destro *et al.* (1973 ▶); Fun *et al.* (2010 ▶); Ghosh *et al.* (1993 ▶); Iball & Low (1974 ▶); Srivastava (1964 ▶); Zhou *et al.* (2004 ▶, 2005 ▶, 2007 ▶). 
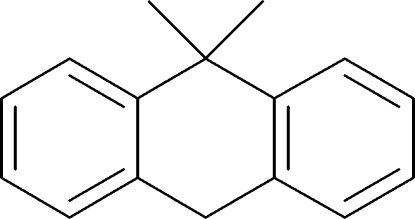


## Experimental

### 

#### Crystal data

C_16_H_16_
*M* *_r_* = 208.29
Monoclinic, 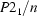

*a* = 12.7042 (15) Å
*b* = 7.4882 (7) Å
*c* = 13.177 (2) Å
β = 107.787 (14)°
*V* = 1193.7 (3) Å^3^
*Z* = 4
Mo *K*α radiation
μ = 0.07 mm^−1^
*T* = 173 K
0.38 × 0.32 × 0.25 mm

#### Data collection

Oxford Xcalibur Eos Gemini diffractometer
Absorption correction: multi-scan (*CrysAlis RED*; Oxford Diffraction, 2010 ▶) *T*_min_ = 0.976, *T*_max_ = 0.984
10733 measured reflections
2958 independent reflections
2447 reflections with *I* > 2σ(*I*)
*R*_int_ = 0.023

#### Refinement

*R*[*F* ^2^ > 2σ(*F* ^2^)] = 0.050
*wR*(*F* ^2^) = 0.144
*S* = 1.01
2958 reflections
147 parameters
H-atom parameters constrained
Δρ_max_ = 0.19 e Å^−3^
Δρ_min_ = −0.21 e Å^−3^

### 

Data collection: *CrysAlis PRO* (Oxford Diffraction, 2010 ▶); cell refinement: *CrysAlis PRO*; data reduction: *CrysAlis RED* (Oxford Diffraction, 2010 ▶); program(s) used to solve structure: *SHELXS97* (Sheldrick, 2008 ▶); program(s) used to refine structure: *SHELXL97* (Sheldrick, 2008 ▶); molecular graphics: *SHELXTL* (Sheldrick, 2008 ▶); software used to prepare material for publication: *SHELXTL*.

## Supplementary Material

Crystal structure: contains datablock(s) global, I. DOI: 10.1107/S1600536811033526/bt5616sup1.cif

Structure factors: contains datablock(s) I. DOI: 10.1107/S1600536811033526/bt5616Isup2.hkl

Supplementary material file. DOI: 10.1107/S1600536811033526/bt5616Isup3.cml

Additional supplementary materials:  crystallographic information; 3D view; checkCIF report

## Figures and Tables

**Table 1:** Hydrogen-bond geometry (Å, °) *Cg*3 is the centroid of the C8–C13 benzene ring.

*D*—H⋯*A*	*D*—H	H⋯*A*	*D*⋯*A*	*D*—H⋯*A*
C10—H10*A*⋯*Cg*3^i^	0.95	2.75	3.7072 (16)	177

Symmetry code: (i) 
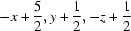
.

**Fig. 1.**
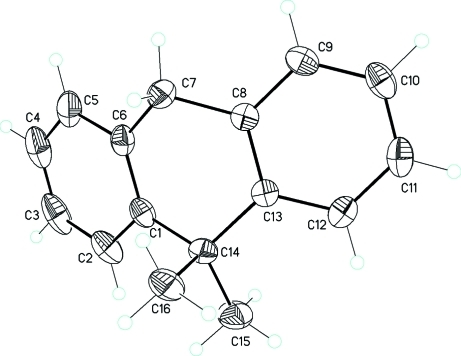
Molecular structure of the title co-crystal salt showing the atom labeling scheme and 50% probability displacement ellipsoids.

**Fig. 2.**
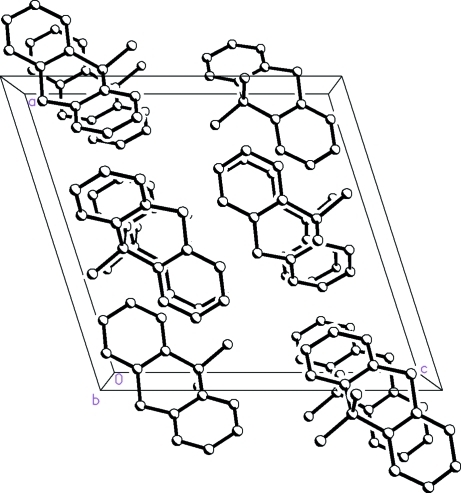
Packing diagram of the title co-crystal salt viewed down the b axis.

## supplementary crystallographic information

### Comment

Anthracene and its derivatives are long known polycyclic aromatic compounds showing a high potential for use in materials science (*e.g.* fluorescence probing, photochromic systems, electroluminescence) and several reviews have been published. Anthrone is a tricyclic aromatic hydrocarbon which is used for a popular cellulose assay and in the colorometric determination of carbohydrates (Trevelyan, 1952) and anthracene itself is used in the production of red dye alizarin. The crystal structures of anthrone (Srivastava, 1964), 10-bromoanthrone (Destro *et al.*, 1973), 9,10-dimethylanthracene (Iball & Low, 1974), benzylideneanthrone at 193 K (Ghosh *et al.*, 1993), 10-(2-methylbenzylidene)anthrone (Zhou *et al.*, 2004), 10-(3,4-dimethoxybenzylidene)anthrone (Zhou *et al.*, 2005), 10-(4-hydroxy-3-nitrobenzylidene)anthrone (Zhou *et al.*, 2007) and 10,10-dimethylanthrone (Fun *et al.*, 2010) have been reported. In view of the importance of anthracene derivatives, this paper reports the crystal structure of the title compound, (I), C_16_H_16_.

In the title compound, C_16_H_16_, the center benzene ring (C1/C6–C8/C13/C14) with puckering parameters, Q, θ, φ, of 0.4930 (13) Å, 92.27 (15)°, 120.13 (15)°, adopts a boat conformation with a dihedral angle of 34.7 (9)° between the mean planes of the two fused benzene rings (Fig. 1). The two methyl groups at the apex of the center benzene ring are in axial and equatorial conformations. The crystal packing is stabilized by weak C—H···*Cg*π-ring intermolecular interactions (Fig. 2).

### Experimental

The title compound was obtained as a gift sample from R. L. Fine Chemicals, Bangalore. X-ray quality crystals were grown from toluene solution by slow evaporation (320–322 K).

### Refinement

All of the H atoms were placed in their calculated positions and then refined using the riding model with C—H lengths of 0.95 Å (CH), 0.99 Å (CH) or 0.98 Å (CH_3_). The isotropic displacement parameters for these atoms were set to 1.19–1.20 (CH), 1.2 (CH_2_) or 1.49 (CH_3_) times *U*_eq_ of the parent atom.

### Crystal data

**Table:**

C_16_H_16_	*F*(000) = 448
*M**_r_* = 208.29	*D*_x_ = 1.159 Mg m^−^^3^
Monoclinic, *P*2_1_/*n*	Mo *K*α radiation, λ = 0.71073 Å
Hall symbol: -P 2yn	Cell parameters from 4436 reflections
*a* = 12.7042 (15) Å	θ = 3.3–32.2°
*b* = 7.4882 (7) Å	µ = 0.07 mm^−^^1^
*c* = 13.177 (2) Å	*T* = 173 K
β = 107.787 (14)°	Block, colourless
*V* = 1193.7 (3) Å^3^	0.38 × 0.32 × 0.25 mm
*Z* = 4	

### Data collection

**Table:**

Oxford Xcalibur Eos Gemini diffractometer	2958 independent reflections
Radiation source: Enhance (Mo) X-ray Source	2447 reflections with *I* > 2σ(*I*)
graphite	*R*_int_ = 0.023
Detector resolution: 16.1500 pixels mm^-1^	θ_max_ = 28.3°, θ_min_ = 3.4°
ω scans	*h* = −16→16
Absorption correction: multi-scan (*CrysAlis RED*; Oxford Diffraction, 2010)	*k* = −9→9
*T*_min_ = 0.976, *T*_max_ = 0.984	*l* = −17→17
10733 measured reflections	

### Refinement

**Table:**

Refinement on *F*^2^	Primary atom site location: structure-invariant direct methods
Least-squares matrix: full	Secondary atom site location: difference Fourier map
*R*[*F*^2^ > 2σ(*F*^2^)] = 0.050	Hydrogen site location: inferred from neighbouring sites
*wR*(*F*^2^) = 0.144	H-atom parameters constrained
*S* = 1.01	*w* = 1/[σ^2^(*F*_o_^2^) + (0.0778*P*)^2^ + 0.2041*P*] where *P* = (*F*_o_^2^ + 2*F*_c_^2^)/3
2958 reflections	(Δ/σ)_max_ < 0.001
147 parameters	Δρ_max_ = 0.19 e Å^−^^3^
0 restraints	Δρ_min_ = −0.21 e Å^−^^3^

### Special details

**Table:**

Geometry. All e.s.d.'s (except the e.s.d. in the dihedral angle between two l.s. planes) are estimated using the full covariance matrix. The cell e.s.d.'s are taken into account individually in the estimation of e.s.d.'s in distances, angles and torsion angles; correlations between e.s.d.'s in cell parameters are only used when they are defined by crystal symmetry. An approximate (isotropic) treatment of cell e.s.d.'s is used for estimating e.s.d.'s involving l.s. planes.
Refinement. Refinement of *F*^2^ against ALL reflections. The weighted *R*-factor *wR* and goodness of fit *S* are based on *F*^2^, conventional *R*-factors *R* are based on *F*, with *F* set to zero for negative *F*^2^. The threshold expression of *F*^2^ > σ(*F*^2^) is used only for calculating *R*-factors(gt) *etc*. and is not relevant to the choice of reflections for refinement. *R*-factors based on *F*^2^ are statistically about twice as large as those based on *F*, and *R*-factors based on ALL data will be even larger.

### Fractional atomic coordinates and isotropic or equivalent isotropic displacement parameters (Å^2^)

**Table:**

	*x*	*y*	*z*	*U*_iso_*/*U*_eq_	
C1	0.39240 (10)	0.13364 (15)	0.28673 (10)	0.0417 (3)	
C2	0.28419 (11)	0.07383 (19)	0.26861 (15)	0.0597 (4)	
H2A	0.2447	0.0248	0.2012	0.072*	
C3	0.23363 (14)	0.0853 (2)	0.34811 (18)	0.0743 (5)	
H3A	0.1599	0.0440	0.3347	0.089*	
C4	0.28927 (16)	0.1558 (2)	0.44566 (17)	0.0736 (5)	
H4A	0.2541	0.1644	0.4996	0.088*	
C5	0.39589 (14)	0.2141 (2)	0.46526 (13)	0.0605 (4)	
H5A	0.4345	0.2623	0.5331	0.073*	
C6	0.44818 (10)	0.20332 (16)	0.38678 (10)	0.0439 (3)	
C7	0.56538 (11)	0.26594 (18)	0.41022 (9)	0.0465 (3)	
H7A	0.5811	0.3571	0.4673	0.056*	
H7B	0.6160	0.1640	0.4364	0.056*	
C8	0.58672 (9)	0.34389 (15)	0.31365 (9)	0.0361 (3)	
C9	0.66134 (9)	0.48356 (17)	0.32422 (10)	0.0451 (3)	
H9A	0.6981	0.5294	0.3932	0.054*	
C10	0.68302 (10)	0.55675 (18)	0.23709 (12)	0.0518 (3)	
H10A	0.7340	0.6525	0.2454	0.062*	
C11	0.62997 (11)	0.48946 (19)	0.13806 (12)	0.0539 (4)	
H11A	0.6445	0.5386	0.0772	0.065*	
C12	0.55516 (10)	0.35000 (18)	0.12576 (10)	0.0463 (3)	
H12A	0.5192	0.3050	0.0564	0.056*	
C13	0.53167 (8)	0.27457 (15)	0.21306 (8)	0.0352 (3)	
C14	0.45293 (9)	0.11660 (16)	0.20299 (9)	0.0400 (3)	
C15	0.37195 (13)	0.1028 (2)	0.09028 (11)	0.0622 (4)	
H15A	0.3270	0.2114	0.0737	0.093*	
H15B	0.3237	−0.0010	0.0859	0.093*	
H15C	0.4133	0.0890	0.0390	0.093*	
C16	0.52239 (12)	−0.05675 (18)	0.22602 (12)	0.0536 (4)	
H16A	0.5754	−0.0507	0.2978	0.080*	
H16B	0.5624	−0.0698	0.1736	0.080*	
H16C	0.4734	−0.1595	0.2213	0.080*	

### Atomic displacement parameters (Å^2^)

**Table:**

	*U*^11^	*U*^22^	*U*^33^	*U*^12^	*U*^13^	*U*^23^
C1	0.0379 (6)	0.0300 (5)	0.0586 (7)	0.0032 (4)	0.0167 (5)	0.0062 (5)
C2	0.0446 (7)	0.0415 (7)	0.0949 (11)	−0.0046 (6)	0.0242 (7)	0.0008 (7)
C3	0.0569 (9)	0.0455 (8)	0.1372 (17)	0.0005 (7)	0.0545 (11)	0.0164 (10)
C4	0.0899 (12)	0.0467 (8)	0.1113 (15)	0.0097 (8)	0.0710 (12)	0.0189 (9)
C5	0.0819 (10)	0.0480 (8)	0.0647 (9)	0.0075 (7)	0.0419 (8)	0.0132 (7)
C6	0.0509 (7)	0.0366 (6)	0.0482 (6)	0.0054 (5)	0.0210 (5)	0.0103 (5)
C7	0.0496 (7)	0.0496 (7)	0.0363 (6)	0.0001 (6)	0.0070 (5)	0.0027 (5)
C8	0.0306 (5)	0.0364 (6)	0.0394 (6)	0.0052 (4)	0.0080 (4)	0.0013 (4)
C9	0.0353 (6)	0.0404 (6)	0.0560 (7)	0.0008 (5)	0.0086 (5)	−0.0044 (5)
C10	0.0394 (6)	0.0400 (6)	0.0797 (10)	0.0008 (5)	0.0239 (6)	0.0057 (6)
C11	0.0516 (7)	0.0534 (8)	0.0663 (9)	0.0096 (6)	0.0323 (7)	0.0182 (7)
C12	0.0464 (6)	0.0536 (7)	0.0400 (6)	0.0088 (6)	0.0148 (5)	0.0043 (5)
C13	0.0305 (5)	0.0356 (6)	0.0388 (5)	0.0060 (4)	0.0094 (4)	0.0016 (4)
C14	0.0368 (6)	0.0373 (6)	0.0426 (6)	−0.0001 (4)	0.0074 (5)	−0.0036 (5)
C15	0.0559 (8)	0.0667 (10)	0.0526 (8)	−0.0121 (7)	−0.0005 (6)	−0.0092 (7)
C16	0.0547 (7)	0.0363 (6)	0.0711 (9)	0.0043 (6)	0.0210 (7)	−0.0059 (6)

### Geometric parameters (Å, °)

**Table:**

C1—C6	1.3935 (18)	C9—C10	1.3739 (19)
C1—C2	1.3953 (18)	C9—H9A	0.9500
C1—C14	1.5309 (17)	C10—C11	1.369 (2)
C2—C3	1.389 (2)	C10—H10A	0.9500
C2—H2A	0.9500	C11—C12	1.388 (2)
C3—C4	1.370 (3)	C11—H11A	0.9500
C3—H3A	0.9500	C12—C13	1.3933 (16)
C4—C5	1.370 (2)	C12—H12A	0.9500
C4—H4A	0.9500	C13—C14	1.5285 (16)
C5—C6	1.3917 (18)	C14—C15	1.5301 (17)
C5—H5A	0.9500	C14—C16	1.5467 (17)
C6—C7	1.5004 (18)	C15—H15A	0.9800
C7—C8	1.4981 (16)	C15—H15B	0.9800
C7—H7A	0.9900	C15—H15C	0.9800
C7—H7B	0.9900	C16—H16A	0.9800
C8—C9	1.3897 (17)	C16—H16B	0.9800
C8—C13	1.3962 (15)	C16—H16C	0.9800
			
C6—C1—C2	118.15 (13)	C11—C10—C9	118.94 (12)
C6—C1—C14	119.40 (10)	C11—C10—H10A	120.5
C2—C1—C14	122.38 (12)	C9—C10—H10A	120.5
C3—C2—C1	120.64 (16)	C10—C11—C12	120.56 (12)
C3—C2—H2A	119.7	C10—C11—H11A	119.7
C1—C2—H2A	119.7	C12—C11—H11A	119.7
C4—C3—C2	120.46 (15)	C11—C12—C13	121.37 (12)
C4—C3—H3A	119.8	C11—C12—H12A	119.3
C2—C3—H3A	119.8	C13—C12—H12A	119.3
C3—C4—C5	119.77 (15)	C12—C13—C8	117.49 (11)
C3—C4—H4A	120.1	C12—C13—C14	122.80 (11)
C5—C4—H4A	120.1	C8—C13—C14	119.66 (10)
C4—C5—C6	120.68 (16)	C13—C14—C15	111.39 (11)
C4—C5—H5A	119.7	C13—C14—C1	109.41 (9)
C6—C5—H5A	119.7	C15—C14—C1	111.62 (10)
C5—C6—C1	120.30 (13)	C13—C14—C16	108.27 (9)
C5—C6—C7	119.85 (12)	C15—C14—C16	107.92 (11)
C1—C6—C7	119.85 (11)	C1—C14—C16	108.10 (10)
C8—C7—C6	111.87 (10)	C14—C15—H15A	109.5
C8—C7—H7A	109.2	C14—C15—H15B	109.5
C6—C7—H7A	109.2	H15A—C15—H15B	109.5
C8—C7—H7B	109.2	C14—C15—H15C	109.5
C6—C7—H7B	109.2	H15A—C15—H15C	109.5
H7A—C7—H7B	107.9	H15B—C15—H15C	109.5
C9—C8—C13	120.23 (11)	C14—C16—H16A	109.5
C9—C8—C7	120.20 (11)	C14—C16—H16B	109.5
C13—C8—C7	119.57 (10)	H16A—C16—H16B	109.5
C10—C9—C8	121.42 (12)	C14—C16—H16C	109.5
C10—C9—H9A	119.3	H16A—C16—H16C	109.5
C8—C9—H9A	119.3	H16B—C16—H16C	109.5
			
C6—C1—C2—C3	0.6 (2)	C10—C11—C12—C13	0.06 (19)
C14—C1—C2—C3	177.46 (12)	C11—C12—C13—C8	−0.35 (17)
C1—C2—C3—C4	0.1 (2)	C11—C12—C13—C14	−177.76 (11)
C2—C3—C4—C5	−0.6 (2)	C9—C8—C13—C12	0.34 (16)
C3—C4—C5—C6	0.4 (2)	C7—C8—C13—C12	−179.27 (10)
C4—C5—C6—C1	0.3 (2)	C9—C8—C13—C14	177.83 (10)
C4—C5—C6—C7	−179.33 (13)	C7—C8—C13—C14	−1.78 (16)
C2—C1—C6—C5	−0.75 (18)	C12—C13—C14—C15	−22.82 (16)
C14—C1—C6—C5	−177.72 (11)	C8—C13—C14—C15	159.83 (11)
C2—C1—C6—C7	178.86 (12)	C12—C13—C14—C1	−146.71 (11)
C14—C1—C6—C7	1.88 (17)	C8—C13—C14—C1	35.93 (13)
C5—C6—C7—C8	−146.93 (12)	C12—C13—C14—C16	95.69 (13)
C1—C6—C7—C8	33.46 (16)	C8—C13—C14—C16	−81.66 (13)
C6—C7—C8—C9	146.92 (11)	C6—C1—C14—C13	−35.94 (14)
C6—C7—C8—C13	−33.47 (16)	C2—C1—C14—C13	147.22 (12)
C13—C8—C9—C10	−0.04 (18)	C6—C1—C14—C15	−159.70 (12)
C7—C8—C9—C10	179.56 (11)	C2—C1—C14—C15	23.46 (17)
C8—C9—C10—C11	−0.25 (19)	C6—C1—C14—C16	81.76 (13)
C9—C10—C11—C12	0.24 (19)	C2—C1—C14—C16	−95.08 (14)

### Hydrogen-bond geometry (Å, °)

**Table:**

Cg3 is the centroid of the C8–C13 benzene ring.

**Table:**

*D*—H···*A*	*D*—H	H···*A*	*D*···*A*	*D*—H···*A*
C10—H10A···Cg3^i^	0.95	2.75	3.7072 (16)	177

Symmetry codes: (i) −*x*+5/2, *y*+1/2, −*z*+1/2.
